# Methylation-Driven Genes Identified as Novel Prognostic Indicators for Thyroid Carcinoma

**DOI:** 10.3389/fgene.2020.00294

**Published:** 2020-03-31

**Authors:** Liting Lv, Liyu Cao, Guinv Hu, Qinyan Shen, Jinzhong Wu

**Affiliations:** Department of Thyroid and Breast Surgery, Affiliated Dongyang Hospital of Wenzhou Medical University, Dongyang, China

**Keywords:** thyroid carcinoma, methylation-driven genes, biomarker, TCGA, prognostic indicators

## Abstract

**Background:**

Aberrant DNA methylation plays an crucial role in tumorigenesis through regulating gene expression. Nevertheless, the exact role of methylation in the carcinogenesis of thyroid cancer and its association with prognosis remains unclear. The purpose of this study is to explore the DNA methylation-driven genes in thyroid cancer by integrative bioinformatics analysis.

**Methods:**

The transcriptome profiling data and DNA methylation data of thyroid cancer were downloaded from The Cancer Genome Atlas (TCGA) database. The methylmix R package was used to screen DNA methylation-driven genes in thyroid cancer. Gene Ontology (GO) and Kyoto Encyclopedia of Genes and Genomes (KEGG) analysis were conducted to annotate the function of methylation-driven genes. Univariate Cox regression analyses was performed to distinguish prognosis-related methylation-driven genes. Multivariate Cox regression analyses was utilized to build a prognostic multi-gene signature. A survival analysis was carried out to determine the individual prognostic significance of this multi-gene signature.

**Results:**

A total of 51 methylation-driven genes were identified. The functional analysis indicated that these genes were significantly enriched in diverse biological processes (BP) and pathways related to the malignancy processes. Four of these genes (RDH5, TREM1, BIRC7, and SLC26A7) were selected to construct the risk evaluation model. Patients in the low-risk group had an conspicuously better overall survival (OS) than those in high-risk group (*p* < 0.001). The area under the receiver operating characteristic (ROC) curve for this model was 0.836, suggesting a good specificity and sensitivity. Subsequent survival analysis revealed that this four-gene signature served as an independent indicator for the prognosis of thyroid cancer. Moreover, the prognostic signature was well validated in a external thyroid cancer cohort.

**Conclusion:**

We identified methylation-driven genes in thyroid cancer with independent prognostic value, which may offer new insight into molecular mechanisms of thyroid cancer and provide new possibility for individualized treatment of thyroid cancer patients.

## Introduction

The incidence of thyroid cancer has increased rapidly in the United States over the last four decades (Lim et al., 2017; Bray et al., 2018). As the fifth most commonly diagnosed cancer in women, thyroid cancer accounts for 40900 new cases estimated by the latest cancer statistic report in the United States (Siegel et al., 2018). This is driven largely by increasing prevalence of papillary thyroid cancer (PTC) which is identified as the most common and least aggressive histologic type in thyroid cancer (Lim et al., 2017). Although thyroid cancer is considered as an indolent malignancy with favorable prognosis, a few patients may suffer local and/or distant recurrence and metastasis, even after surgery or adjuvant radioactive iodine therapy, leading to a poor prognosis (Leboulleux et al., 2016; Yang et al., 2019). Hence, exploring effective and promising biomarkers capable of distinguishing thyroid cancer patients with worse prognosis is of important clinical significance, which is in huge demand.

DNA methylation, a critical element in epigenetic modifications, plays a vital role in the transcriptional regulation and maintains the genome stability (Pu et al., 2016). Accumulating evidence have indicated that aberrant DNA methylation occurred on CpG islands of promoters is capable of regulating expression of many tumor-associated genes and is critical for cancer development (Ferry et al., 2017; Zheng et al., 2017). It was well demonstrated that hypomethylation of oncogene or hypermethylation of tumor suppressor acts as crucial initial events in carcinogenesis (Huang et al., 2011; Yang et al., 2017). For instance, Gao et al. (2017) demonstrated that methylation of TMEM176A was associated with human colorectal cancer development. Aberrant promoter methylation of SOCS-1 was proved to contribute to the pathogenesis of hepatocellular carcinoma (Zhao et al., 2016).

DNA methylation can also be utilized to diagnose cancer as well as predict clinical outcomes. For example, a panel of DNA methylation biomarkers were validated by Lasseigne et al. (2014) to have a good performance in early clinical detection of renal cell carcinoma. Casadio et al. reported that the detection of the methylation frequencies of GSTP1, HIC1, and RASSF1A could be used to predict recurrence of bladder cancer (Casadio et al., 2013). Hence, studying DNA methylation may help elucidate the mechanism of cancer development as well as explore promising diagnostic and prognostic biomarker. Recently, a computational protocol called MethylMix, an algorithm implemented in the R program, can be utilized to identify disease specific hyper/hypomethylation genes significantly associated with their expression (methylation-driven genes) (Cedoz et al., 2018). Several studies focusing on identifying DNA methylation-driven genes using the MethylMix algorithm have been reported in various cancer, such as lung adenocarcinoma, hepatocellular carcinoma, prostate adenocarcinoma. Nevertheless, there is still lack of studies on assessing methylation-driven genes in thyroid carcinoma.

In this study, we used an integrative approach to identify thyroid carcinoma related methylation-driven genes by combining the transcriptomic and DNA methylation profiles from the TCGA database. A four methylation-driven signatures was successfully identified by constructing a Cox survival predictive model, which could effectively distinguish thyroid carcinoma patients with bad prognosis. Our findings will provide new insights into the molecular mechanisms of thyroid carcinoma and prompt a more individualized therapies for this prevalent disease.

## Materials and Methods

### Data Acquisition and Preprocessing

The available RNA-seq transcriptome data, DNA methylation data, and clinicopathological information of thyroid carcinoma were downloaded from the TCGA database^1^. There were 567 samples with gene transcriptome data (58 normal and 509 tumor), 570 samples with DNA methylation data (56 normal and 514 tumor), and 506 patients with available survival data.

The differentially expressed genes (DEGs) between tumor and normal samples were screened firstly by utilizing the “limma” R package. The cutoff criteria was set as | log2 fold change (FC)| > 1 and *p* < 0.05.

### Screening for DNA Methylation-Driven Genes

The “MethylMix” R package was conducted for screening the DNA methylation-driven genes in thyroid carcinoma by integrating DNA methylation data and paired gene expression data. The cutoff criteria was set as | log2 FC| > 0, *p* < 0.05 and correlation coefficient < −0.3. In brief, genes of which the expression was significantly affected by the corresponding DNA methylation events were chosen for further analysis. Then, a beta mixture model was constructed for defining the degree of methylation across the large samples. Finally, Wilcoxon rank test was utilized to calculate differential methylation in tumor and normal samples, and genes met the cutoff criteria were considered as DNA methylation-driven gene (Cedoz et al., 2018). The expression and methylation pattern of those DNA methylation-driven genes in thyroid carcinoma were visualized by “pheatmap” R package.

### Functional Enrichment Analysis

Gene ontology (GO) enrichment analysis were conducted to annotate those identified DNA methylation-driven genes by utilizing the Database of Annotation, Visualization and Integrated Discovery (DAVID) v6.8^2^. The top significantly enriched (*p* < 0.05) GO terms of biological process (BP) were visualized by “GOplot” R package. Subsequently, the Kyoto Encyclopedia of Genes and Genomes (KEGG) analysis was also used to perform the pathway enrichment analysis for those DNA methylation-driven genes through KOBAS 3.0^3^. The “GOplot” R package was also used to visualize the significantly enriched pathways (*p* < 0.05).

Gene Set Enrichment Analysis (GSEA) was performed using the software GSEA v4.0.3^4^. After normalization of gene expressions data of 567 samples (509 tumor and 58 normal), GO analysis was conducted by employment of GSEA software mentioned above. GO gene sets from MSigDB (Molecular Signature Database) were used as reference. The analysis process was repeated 1,000 times using the default weighted enrichment statistics methods. All other parameters were set based on their default values.

### Construction of Prognostic Signatures and Survival Analysis

Univariate Cox regression analysis was conducted to determine the relationship between the expression of DNA methylation-driven genes and prognosis of thyroid carcinoma patients. Genes with a *p*-value < 0.05 were regarded as prognostic methylation-driven genes and were subsequently fitted into the multivariate Cox regression analysis. A DNA methylation-driven gene-based prediction model was constructed by the linear combination of the expression levels of methylation-driven genes using coefficients (β) calculated from multivariate Cox regression as the weights. The risk score for each patient was calculated based on the risk score formula: risk score = expression of gene1 × β1 + expression of gene2 × β2 + …expression of genen × βn. After that, patients were divided into high-risk and low-risk groups by setting the median value of risk scores as cut-off value. The overall survival (OS) of these two groups was calculated by the Kaplan-Meier method with log-rank test. Receiver operating characteristic (ROC) curve were performed to assess the predictive performance of the prognostic model. The expression patterns of DNA methylation-driven genes in this prognostic model were visualized by “pheatmap” R package.

Univariate and multivariate Cox regression analyses were conducted to determine whether the risk score calculated from the prognostic model was independent prognostic factors for thyroid carcinoma patients after considering other clinical features, including age, gender and AJCC stage.

### Cell Culture

The papillary thyroid carcinoma cell lines (TPC-1 and K1) were cultured in RPMI 1640 medium (Gibco, Life Technologies, CA, United States) with 10% fetal bovine serum (Biological Industries, CT, United States) at 37°C and 5% CO_2_.

### The Validation Patient Cohort

The validation study was approved by the First Affiliated Hospital of Zhejiang University. A total of 200 specimens of thyroid cancer were included in the validation cohort. The detailed clinicopathological information of the validated cohort was summarized in Supplementary Table S1. Written informed consent was obtained from all the patients.

### Quantitative Reverse Transcription PCR

The total RNA was extracted using Trizol reagent (Invitrogen, United States). The cDNA for each cell line and tissue specimens was reverse transcribed using the PrimeScript 1st Strand cDNA Synthesis Kit (TaKaRa, Dalian, China). qRT-PCR analysis was conducted using the SYBR-Green method according to standard protocols. The sequences of the primers used were as follows: RDH5, 5′-TGGGTGGAGATGCACGTTAAG-3′ (forward), 5′-GTGTGGGTCCGATGATACCAG-3′ (reverse); TREM1, 5′-GAACTCCGAGCTGCAACTAAA-3′ (forward), 5′-TCTAGCGT GTAGTCACATTTCAC-3′ (reverse); BIRC7, 5′-GCTCTGAGG AGTTGCGTCTG-3′ (forward), 5′-CACACTGTGGACAAAGT CTCTT-3′ (reverse); SLC26A7, 5′-AGAAGGCGACTGCCCA TTTT-3′ (forward), 5′-ACTGCCAACATTATCCCAGACA-3′ (reverse).

#### Validation of the Prognostic Signature

The validated cohort was divided into high-risk group and low-risk group based on their risk scores. The cut-off value was set as the median of the risk scores in TCGA thyroid cancer cohort. Then, the difference of OS between high-risk and low-risk group was calculated. Univariate and multivariate Cox regression analyses were utilized to determine whether risk score was an independent prognostic factor.

### Statistical Analysis

All data in the present study were analyzed by utilizing the R statistical package (R version 3.6.1) unless otherwise stated. A two-tailed *p* < 0.05 was considered statistically significant. ^∗^*p* < 0.05, ^∗∗^*p* < 0.01, and ^∗∗∗^*p* < 0.001.

## Results

### Identification of Methylation-Driven Genes in Thyroid Carcinoma

**FIGURE 1:**
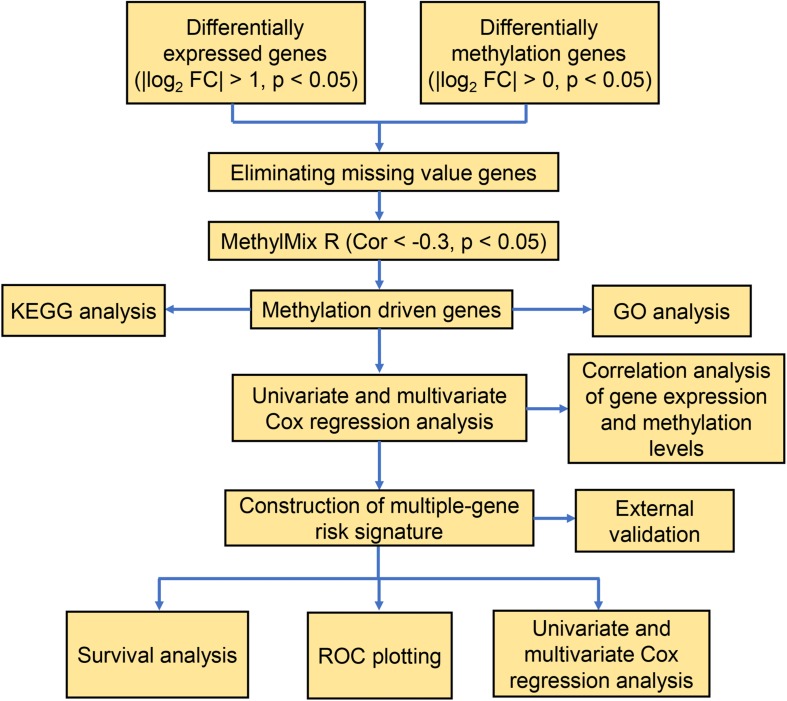
A flow chart of the exploration of methylation-driven genes in thyroid cancer.

**FIGURE 2:**
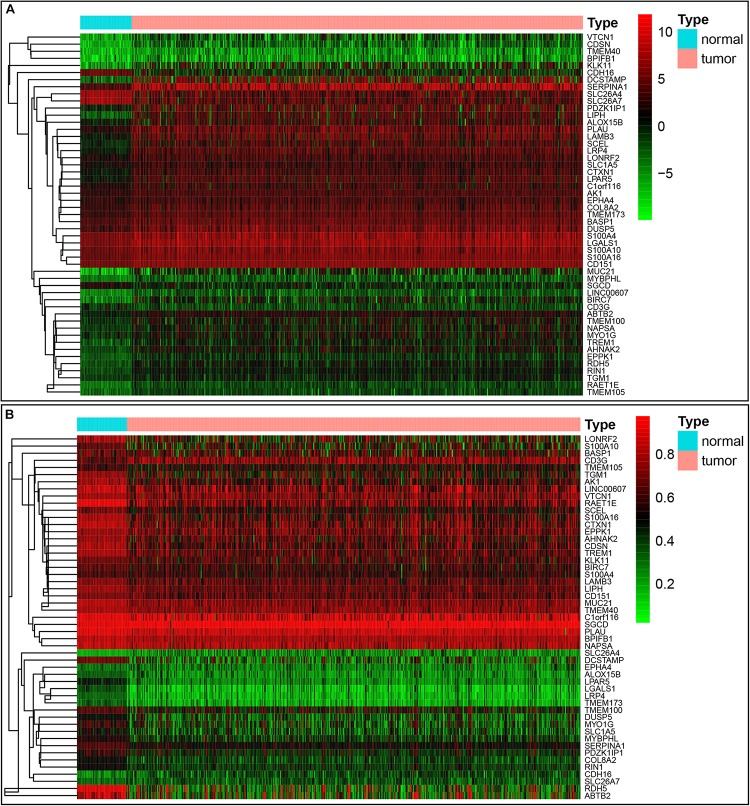
Heatmap of 51 methylation-driven genes in thyroid cancer. **(A)** The expression pattern of 51 methylation-driven genes. Red represents upregulated genes and green represents downregulated genes between tumor and normal tissues. **(B)** The methylation pattern of 51 methylation-driven genes. Red represents highly methylated genes and green represents low methylated genes between tumor and normal tissues.

Our study included RNA-sequencing data from 567 samples from thyroid carcinoma patients, including 58 normal samples and 509 tumor samples. DNA methylation data were extracted from 570 thyroid carcinoma specimens, including 56 normal samples and 514 tumor samples. Using the cutoff criteria of FDR < 0.05 and |log2FC| > 1, a total of 3430 DEGs (1751 upregulated and 1679 downregulated) were screened for further analysis. The gene expression data and DNA methylation data for 3430 DEGs were included in the MethylMix analysis with a screening criteria set as |log2FC| > 0, *p* < 0.05 and Cor < −0.3. As a result, we totally identified 51 DNA methylation-driven genes of which 46 were hypomethylated while 5 were hypermethylated (Table 1). A flow chart of the exploration of methylation-driven genes was shown in Figure 1. The expression pattern and methylation value of methylation-driven genes were shown as heat map (Figures 2A,B).

**TABLE 1:** Methylation-driven genes.

genes	Normal mean	Tumor mean	log FC	*p*-value	Cor	Cor *p*-value
RAET1E	0.914157	0.697235	–0.390797	4.45E−29	–0.534397	6.26E−39
SERPINA1	0.632612	0.514767	–0.297402	1.51E−26	–0.535371	4.32E−39
LPAR5	0.457776	0.273762	–0.741721	3.01E−26	–0.646354	1.54E−61
RDH5	0.898498	0.430300	–1.062172	1.02E−24	–0.644569	4.21E−61
TREM1	0.795173	0.638780	–0.315949	2.06E−24	–0.342375	1.91E−15
LIPH	0.768724	0.665275	–0.208516	2.95E−24	–0.603783	7.03E−52
LRP4	0.325453	0.191412	–0.765766	7.06E−24	–0.494515	9.51E−33
COL8A2	0.496770	0.384904	–0.368082	8.23E−24	–0.442363	8.42E−26
DCSTAMP	0.682648	0.386206	–0.821769	8.25E−22	–0.653196	3.09E−63
LGALS1	0.376251	0.190589	–0.981231	9.00E−22	–0.477586	2.32E−30
TMEM173	0.336766	0.205043	–0.715818	6.24E−21	–0.764352	1.11E−98
MUC21	0.824024	0.723442	–0.187809	1.78E−20	–0.333290	1.14E−14
CTXN1	0.831012	0.659250	–0.334044	3.22E−20	–0.599326	5.96E−51
LONRF2	0.790843	0.531867	–0.572325	5.67E−20	–0.327829	3.23E−14
AHNAK2	0.765057	0.615665	–0.313421	6.59E−20	–0.714908	8.18E−81
AK1	0.775374	0.590682	–0.392510	9.14E−20	–0.493644	1.27E−32
ABTB2	0.810591	0.447253	–0.857882	1.79E−18	–0.678615	5.92E−70
CDSN	0.798050	0.618537	–0.367620	2.49E−18	–0.498284	2.68E−33
SCEL	0.699450	0.568227	–0.299754	9.04E−18	–0.480317	9.76E−31
S100A16	0.794216	0.607415	–0.386850	2.26E−17	–0.547830	3.33E−41
PLAU	0.904954	0.853874	–0.083821	7.95E−17	–0.478322	1.84E−30
CDH16	0.262682	0.383591	0.546251	1.06E−16	–0.393862	2.46E−20
TMEM40	0.797271	0.733412	–0.120446	1.47E−16	–0.465595	9.50E−29
MYBPHL	0.542402	0.425328	–0.350787	1.80E−15	–0.482369	5.07E−31
PDZK1IP1	0.577887	0.459305	–0.331335	2.19E−15	–0.504215	3.54E−34
BPIFB1	0.832361	0.785808	–0.083033	7.10E−15	–0.396836	1.20E−20
RIN1	0.510654	0.425439	–0.263393	7.68E−15	–0.640856	3.33E−60
MYO1G	0.588103	0.410445	–0.518881	2.73E−14	–0.664549	3.73E−66
TGM1	0.758261	0.556121	–0.447296	4.46E−14	–0.612998	7.60E−54
ALOX15B	0.341945	0.265716	–0.363879	5.72E−13	–0.438093	2.77E−25
LAMB3	0.728829	0.638443	–0.191021	1.51E−12	–0.674408	8.53E−69
TMEM100	0.638705	0.506043	–0.335889	1.58E−12	–0.523246	4.06E−37
CD3G	0.635594	0.735447	0.210517	2.10E−12	–0.453344	3.63E−27
LINC00607	0.847477	0.699606	–0.276632	3.24E−12	–0.474113	6.91E−30
SLC26A4	0.187955	0.269248	0.518549	1.25E−11	–0.577639	1.24E−46
SLC1A5	0.506373	0.378163	–0.421193	1.52E−11	–0.548142	2.94E−41
DUSP5	0.493559	0.374514	–0.398205	4.39E−11	–0.513583	1.33E−35
BIRC7	0.655324	0.584382	–0.165297	6.04E−10	–0.480075	1.05E−30
S100A10	0.657108	0.510199	–0.365071	9.06E−10	–0.705123	1.01E−77
CD151	0.714099	0.649581	–0.136613	1.44E−09	–0.484509	2.54E−31
VTCN1	0.785455	0.709718	–0.146284	2.22E−09	–0.492672	1.76E−32
NAPSA	0.847424	0.788968	–0.103117	6.69E−08	–0.608248	7.99E−53
KLK11	0.684627	0.610142	–0.166173	1.74E−07	–0.740069	2.14E−89
EPPK1	0.765503	0.683879	–0.162667	2.20E−07	–0.507768	1.03E−34
SGCD	0.911930	0.923126	0.017604	2.58E−05	–0.465736	9.10E−29
BASP1	0.656438	0.574277	–0.192911	4.44E−05	–0.323740	6.96E−14
S100A4	0.613862	0.580597	–0.080377	9.76E−05	–0.604334	5.39E−52
SLC26A7	0.322965	0.356954	0.144364	0.000287214	–0.408232	7.30E−22
TMEM105	0.596893	0.545987	–0.128605	0.000421052	–0.482211	5.33E−31
C1orf116	0.903649	0.830643	–0.121534	0.014746146	–0.332451	1.34E−14
EPHA4	0.305452	0.293696	–0.056623	0.026836525	–0.507321	1.21E−34

*log FC, log fold change; Cor, Correlation.*

### Functional Analysis of Methylation-Driven Genes in Thyroid Carcinoma

**FIGURE 3:**
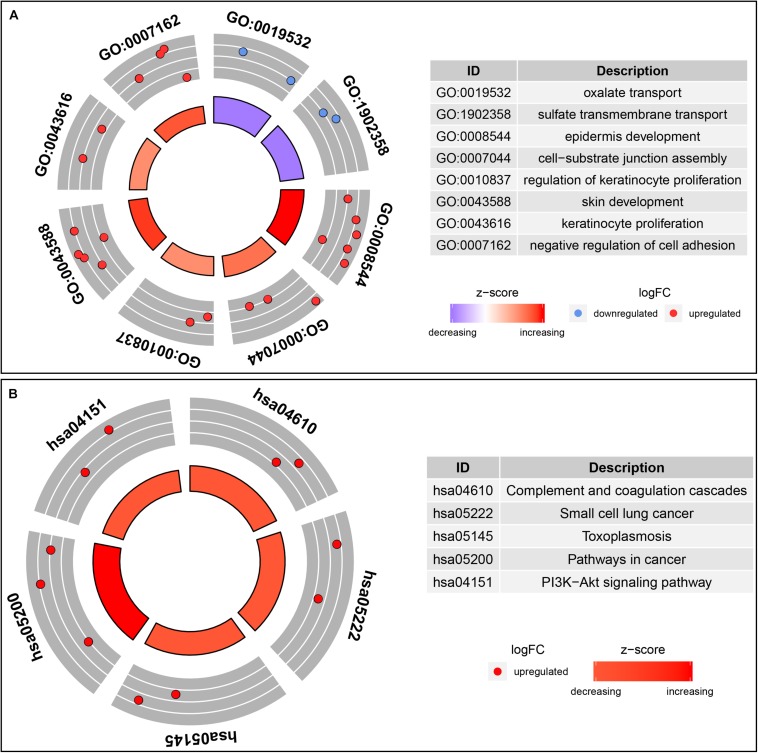
Functional enrichment analysis of methylation-driven genes in thyroid cancer. **(A)** GO enrichment analysis. **(B)** KEGG enrichment analysis. The color of inner circle represents *z* score while the band thickness of inner circle represents the significance of GO terms (log10-adjusted *p* values). The outer circle represents the expression (log_2_ FC) of methylation-driven mRNAs in each enriched GO (gene ontology) term: red dots indicate upregulated methylation-driven mRNAs while blue dots indicate downregulated methylation-driven mRNAs.

In order to understand the possible function of those DNA methylation-driven genes, the GO functional enrichment analysis and KEGG pathway enrichment analysis were conducted. A BP analysis was mainly performed in GO analysis. The result indicated that DNA methylation-driven genes were significantly enriched (*p* < 0.05) in terms associated with cell proliferation, including epidermis development, regulation of keratinocyte proliferation, skin development, keratinocyte proliferation (Figure 3A). In addition, a GO terms with regard to negative regulation of cell adhesion was also statistically significant (*p* < 0.05) (Figure 3A). Moreover, pathway analysis also showed that the methylation-driven genes were significantly enriched in malignancy-related pathways, such as small cell lung cancer, pathways in cancer, and PI3K-Akt signaling pathway. The pathway analysis was shown in Figure 3B. In addition, the GSEA analysis of 51 DNA methylation-driven genes was also performed. The results showed that regulation of wnt signaling pathway, cell signaling, endoplasmic reticulum part, and molecular function regulator were significantly enriched for those DNA methylation-driven genes (Supplementary Figures S1A–D).

### Construction of a Methylation-Driven Gene-Based Risk Signature

**FIGURE 4:**
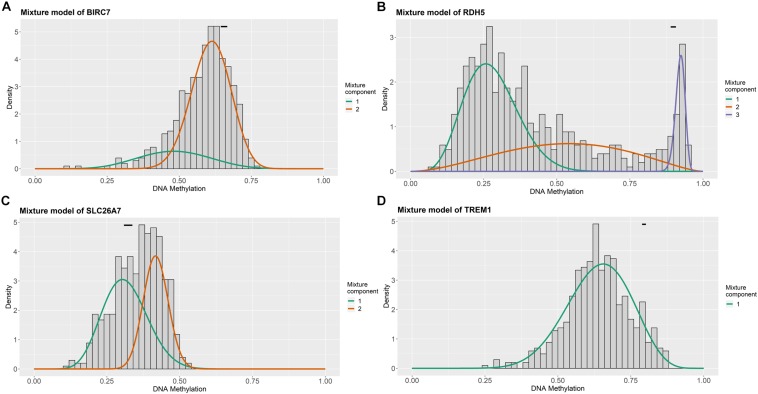
Distribution map of the methylation degree of BIRC7 **(A)**, RDH5 **(B)**, SLC26A7 **(C)**, and TREM1 **(D)** in the risk model. *X*-axis represents the degree of methylation and *Y*-axis represents the number of methylated samples; The black horizontal line represents the methylation degree distribution in the normal samples.

**FIGURE 5:**
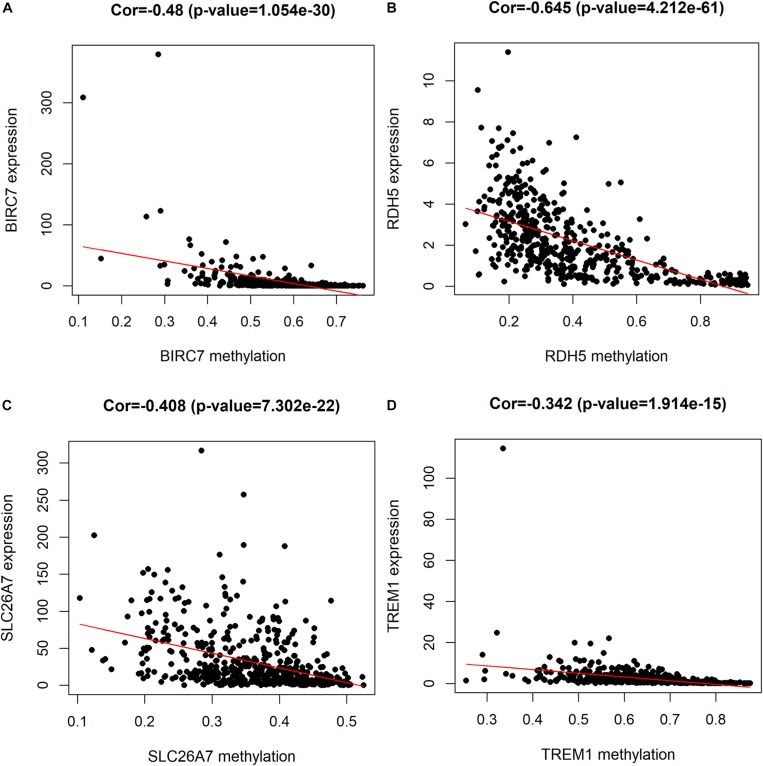
Correlation between the expression and methylation degree of BIRC7 **(A)**, RDH5 **(B)**, SLC26A7 **(C)**, and TREM1 **(D)** in the risk model. *X*-axis represents the methylation degree and *Y*-axis represents gene expression level.

For the purpose of determining the prognostic role of DNA methylation-driven genes in thyroid carcinoma, univariate Cox regression analysis was performed firstly to identify prognosis associated methylation-driven genes in TCGA cohort. The results indicated that 4 methylation-driven genes (TREM1, CDH16, BIRC7, and SLC26A7) were risky genes with HR > 1, while 3 genes (LPAR5, RDH5, and LIPH) served as protective genes with HR < 1 (Supplementary Table S2). Subsequently, multivariate Cox regression analysis was utilized and showed that four methylation-driven genes (RDH5, TREM1, BIRC7, and SLC26A7) were eventually chosen to build a predictive model. The result of multivariate Cox regression analysis was shown in Table 2. Through linear combination of the expression of the 4 methylation-driven genes, the coefficient of each gene were calculated from the multivariate Cox regression analysis. As a result, a risk score for each patient could be calculated using the following formula: risk score = (−0.331) × expression value of RDH5 + (0.165) × expression value of TREM1 + (0.017) × expression value of BIRC7 + (0.016) × expression value of SLC26A7. The mixed models for the four genes in the prognostic model with regard to the methylation degree in normal and tumor tissues were visualized in Figure 4. As shown in Figure 5, all the methylation degrees of RDH5, TREM1, BIRC7, and SLC26A7 were negatively correlated with corresponding gene expression. To validate the role of methylation in the regulation of the expression of these 4 genes, the thyroid cancer cell lines, TPC-1 and K1, were treated with methylation inhibitor 5-aza. As shown in Supplementary Figure S2, the expression of all these 4 genes was significantly up-regulated in a dose-dependent manner, which further demonstrated it as methylation-driven genes.

**TABLE 2:** Coefficients based on a multivariate Cox regression analysis of four genes.

genes	Coef.	HR	95% CI of HR	*p*-value
RDH5	–0.331	0.719	0.457–1.129	0.151
TREM1	0.165	1.180	1.059–1.314	0.003
BIRC7	0.017	1.018	1.007–1.028	0.001
SLC26A7	0.016	1.017	1.007–1.026	0.001

*HR, hazard ratio.*

### Survival and ROC Curve Analysis

**FIGURE 6:**
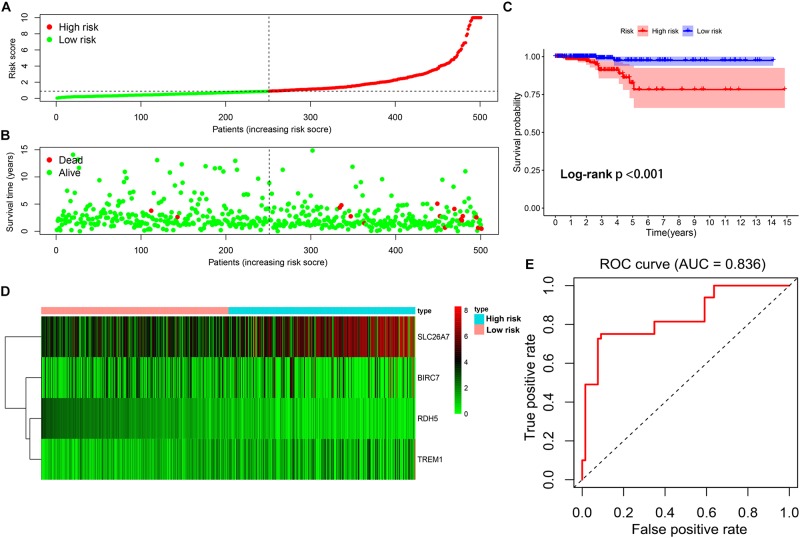
Construction of the multi-gene prognostic signature. **(A)** The distributions of patients’ risk scores. **(B)** The distributions of risk scores and OS status. **(C)** The survival analysis of the two subgroups stratified based on the median of risk scores. **(D)** The expression pattern of the four methylation-driven genes in low- and high-risk groups. **(E)** The ROC curve for evaluating the prediction efficiency of the prognostic signature.

By using the median of risk scores as cut-off value (0.887), a total of 501 thyroid carcinoma patients with complete survival information were divided into the low-risk group (*n* = 251) and high-risk group (*n* = 250). The distributions of the four gene signature-based risk scores were showed in Figure 6A. Moreover, the distributions of risk scores and OS status of each patient were displayed in Figure 6B, suggesting a good discrimination between low-risk and high-risk group. By means of plotting Kaplan-Meier curve, survival analysis demonstrated that patients in the low-risk group had a conspicuously better OS than those in the high-risk group (*p* < 0.001) (Figure 6C). In addition, Figure 6D exhibited the expression pattern of these 4 methylation-driven genes in thyroid carcinoma. Finally, the ROC curve analysis further showed an excellent prediction efficiency with a AUC value equal to 0.836 (Figure 6E).

### The Signature-Based Risk Score Was an Independent Prognostic Factor

**FIGURE 7:**
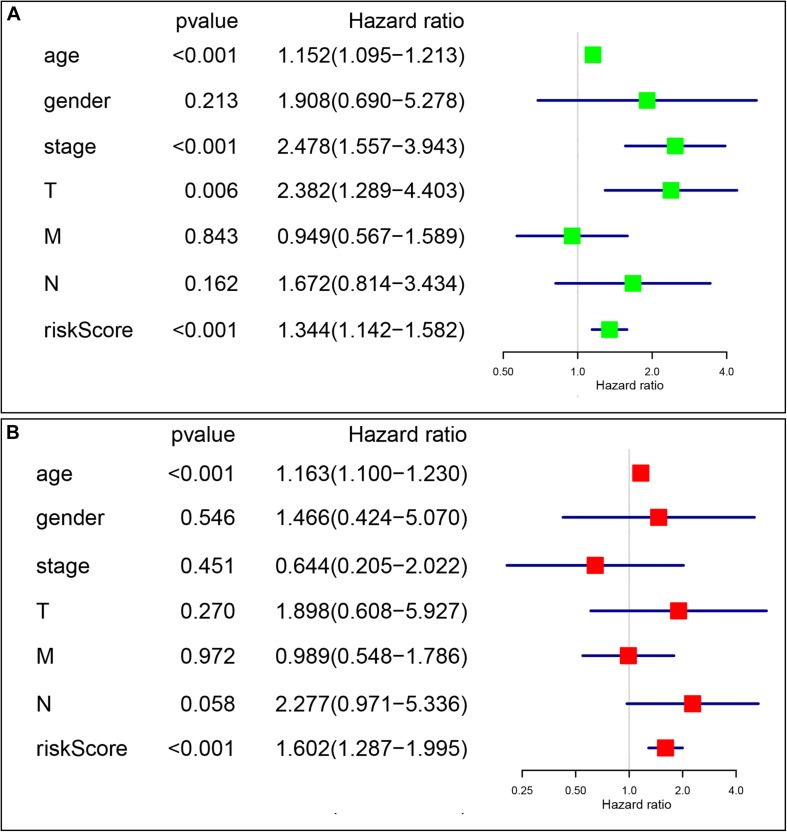
The signature-based risk score was an independent prognostic factor. **(A)** Univariate analysis of the risk score and clinicopathological features in TCGA thyroid cancer cohort. **(B)** Multivariate analysis of the risk score and clinicopathological features in TCGA thyroid cancer cohort.

Univariate and multivariate Cox analyses were conducted to determine if the four-gene signature-based risk score was an independent prognostic factor. The univariate Cox analysis indicated that age (*p* < 0.001, HR = 1.152, 95% CI = 1.095–1.213), AJCC stage (*p* < 0.001, HR = 2.478, 95% CI = 1.557–3.943), T stage (*p* = 0.006, HR = 2.382, 95% CI = 1.289–4.403), and risk score (*p* < 0.001, HR = 1.344, 95% CI = 1.142–1.582) were dramatically associated with the OS (Figure 7A). When all these factors were enlisted into the multivariate Cox regression analysis, only age (*p* < 0.001, HR = 1.163, 95% CI = 1.100–1.230) and risk score (*p* < 0.001, HR = 1.602, 95% CI = 1.287–1.995) were identified as the independent prognostic factors (Figure 7B).

### Validation of the Prognostic Signature

**FIGURE 8:**
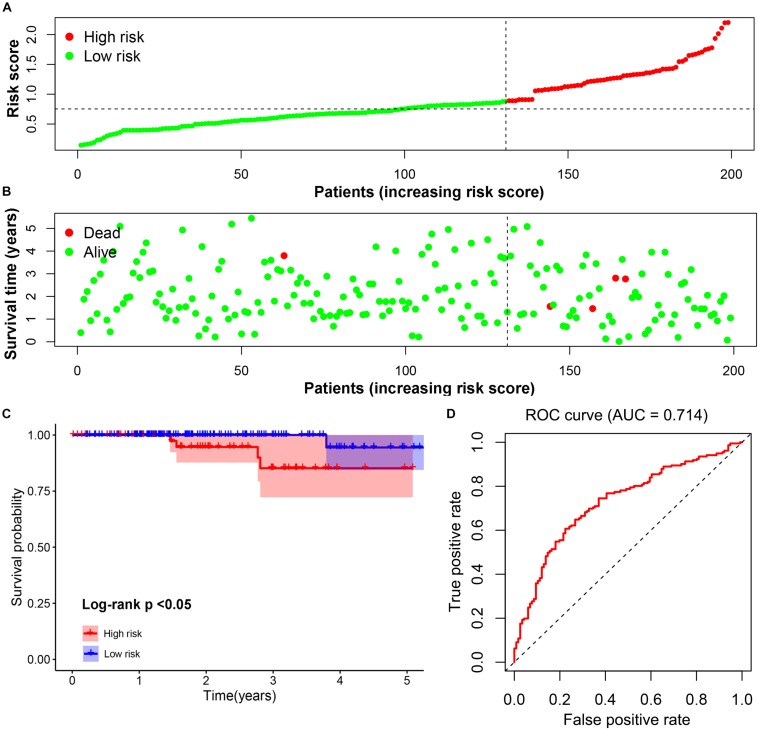
Validation of the prognostic risk signature. **(A,B)** The distributions of prognostic signature-based risk scores. The red dots represent high-risk patients while green dots represent low-risk patients. **(C)** The survival analysis of the two subgroups. **(D)** The ROC curve for evaluating the prediction efficiency of the prognostic signature.

**FIGURE 9:**
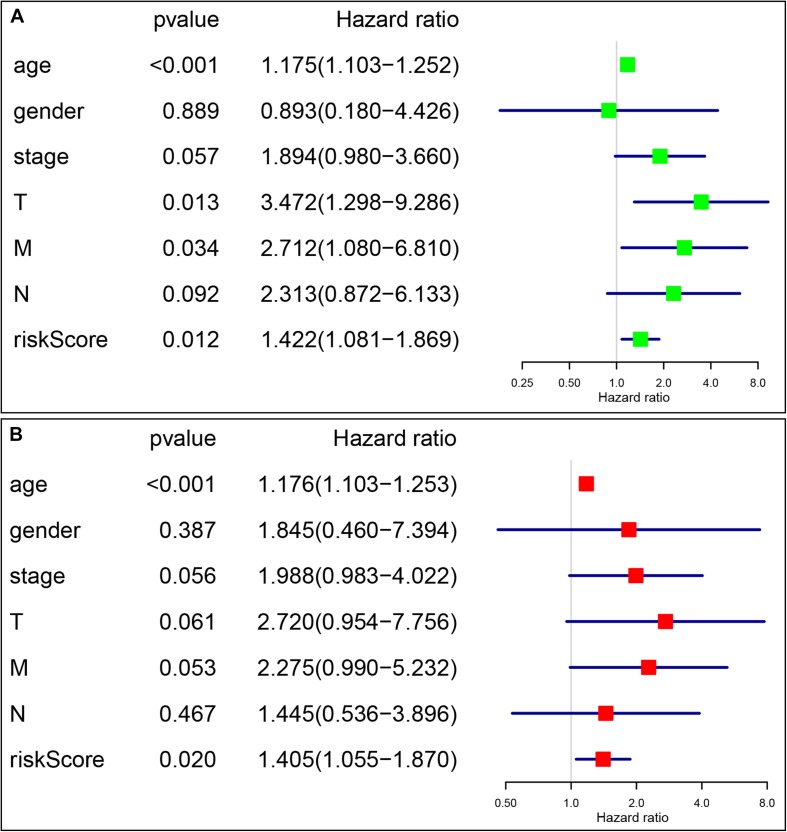
Identification of the independent prognostic factors in the validation group. **(A)** Univariate Cox analyses of the signature based risk score and clinicopathological parameters in validation cohort; **(B)** Multivariate Cox analyses of the signature based risk score and clinicopathological parameters in validation cohort.

The prognostic value of the four-gene risk signature was validated in validation cohort (*n* = 200). Based on aforementioned cut-off value, a total of 68 patients were grouped into high-risk subgroup while the remaining 132 patients were categorized into low-risk group. The distributions of the risk scores and OS status were shown in Figures 8A,B. The Kaplan-Meier curve demonstrated that patients in high risk group had an obviously poorer OS compared to patients with low risk (*p* < 0.05) (Figure 8C). The ROC curves also demonstrated that risk score (AUC = 0.714) had a good predictive ability (Figure 8D). Then, the univariate analysis demonstrated that age (HR = 1.175, 95% CI [1.103–1.252], *p* < 0.001), T stage (HR = 3.472, 95% CI [1.298–9.286], *p* = 0.013), M stage (HR = 2.712, 95% CI [1.080–6.810], *p* = 0.034), and signature-based risk score (HR = 1.422, 95% CI [1.081–1.869], *p* = 0.012) were significantly associated with the OS in validation cohort (Figure 9A). The multivariate analysis further showed that signature-based risk score served as independent prognostic indicators (HR = 1.405, 95% CI [1.055–1.870], *p* = 0.020) (Figure 9B). These results, taken together, convincingly verified the prognostic value of this four-gene risk signature in patients with thyroid cancer.

## Discussion

According to statistics, the overall incidence of thyroid cancer in United States increased significantly from 1994 to 2013 (approximately 3% per year) (Lim et al., 2017). In addition, a dramatically increase in thyroid cancer mortality rate (approximately 1.1% annually), especially in advanced stage PTC (2.9% per year), were also reported in this period (Lim et al., 2017). Indeed, although most of the patients with thyroid cancer have excellent prognosis, part of them have worse prognosis due to tumor recurrence or distant metastasis (Kazaure et al., 2012). Therefore, it is of great importance to excavate novel biomarkers that can indicate these patients with bad prognosis.

A great deal of research have demonstrated a strong relationship between epigenetic aberrations and genetic aberrations in tumorigenesis (You and Jones, 2012; Chang et al., 2013). It is commonly believed that epigenetic changes, such as DNA methylation, can drive abnormal gene expression of crucial genes involved in the development and progression of cancer, including prostate cancer (Chen et al., 2014), liver cancer (Feitelson, 2006), head and neck cancer (Worsham et al., 2014), etc. In addition, previous studies also reported that the methylation status of specific genes significantly associated with worse prognosis (Lee et al., 2006; Zhu et al., 2017). Therefore, the methylation-driven genes could serve as attractive prognostic indicator in tumor patients. For example, Long et al. used two DNA methylation-driven genes, SPP1 and LCAT, to construct two-gene signature which acted as an independent predictor for prognosis of liver cancer (Long et al., 2019). Methylated hub genes, including HOXD3, LAT, and NFE2L3, were proved to be a novel prognostic indicators in clear cell renal cell carcinoma (Wang et al., 2019). However, to our best knowledge, there is still a lack of research on screening DNA methylation-driven genes as prognostic biomarker in thyroid cancer.

In our study, we conducted a comprehensive view of DNA methylation-driven genes in thyroid cancer and developed a prognostic signature based on the expression values of four methylation-driven genes. A cohort of 51 DNA methylation-driven genes was identified firstly in thyroid cancer. The functional analysis indicated that these genes were significantly enriched in diverse BP and pathways ranging from cell proliferation, cell adhesion and pathways in cancer. These results implied that DNA methylation might functionally relate to the malignancy processes of thyroid cancer. Subsequently, a risk multi-genes signature including four methylation-driven genes (RDH5, TREM1, BIRC7, and SLC26A7) was constructed to serve as a reliable predictor by means of univariate and multivariate Cox analysis. Based on the risk score calculated from the four-genes signature, the thyroid cancer patients in TCGA cohort could be divided into two groups with high- or low-risk. Survival analysis indicated that thyroid cancer patients with high risk had significantly inferior OS than those with low risk. The AUC of the ROC curve based on this signature was as high as 0.836 at 5 years of OS. The results of univariate and multivariate Cox analyses further demonstrated that signature-based risk score was an independent prognostic factor. Finally, we validated this four-gene risk signature in validation cohort, which further suggested its convincing prognostic value in thyroid cancer patients. Interesting, we found that traditional pathological indicators, such as AJCC stage, was no longer independent prognostic factors, implying that our risk signature could emerge as a stable and reliable indicator capable of predicting the prognosis of thyroid cancer. Although we are unable to confirm whether the DNA-methylation driven genes based risk factor can perform better than other biomarkers, such as DNA-methylation driven genes and variants/fusions, the prognostic signature built in our study could contribute to distinguish thyroid cancer patients with poor outcome. Importantly, we hold the opinion that for those thyroid cancer patients classified as high-risk, an aggressive transdisciplinary management, such as surgery and adjuvant radioactive iodine, should be considered.

Among the four methylation-driven genes, the high expression level of TREM1, BIRC7, and SLC26A7 prognosticated low survival rate, whereas RDH5 acted as protective genes to suggest good prognosis of thyroid cancer. TREM1 is a activating member of the Ig superfamily that functions as a potent amplifier of pro-inflammatory innate immune responses (Bouchon et al., 2000). Mounting evidence indicated that the overexpression of TREM1 is associated with the development of several types of cancer, such as colorectal carcinoma (Saurer et al., 2017) and hepatocellular tumor (Duan et al., 2015). In terms of BIRC7, Ge et al. (2019) revealed that BIRC7, an important member of the human inhibitor of apoptosis proteins (IAPs) family, promoted colon cancer progression. Wei et al. (2018) also reported that the hypomethylation of BIRC7 was closely related to the pathogenesis of bone tumor. Meanwhile, overexpression of BIRC7 was also used to predict the worse prognosis of various cancer patients (Ibrahim et al., 2014; Sun et al., 2018). Consistently, our study demonstrated that the hypomethylation of TREM1 and BIRC7 contributed to its overexpression, suggesting its potential protumorigenic role in thyroid cancer. In addition, SLC26A7 and RDH5 also have been previously identified to be associated with anaplastic thyroid carcinoma (ATC) and papillary thyroid carcinoma, respectively (Beltrami et al., 2017; Weinberger et al., 2017). Therefore, our integrative analysis provided a convincing clue that genes potentially regulated by DNA methylation may serve as potential drivers and biomarkers related to thyroid cancer development. Our findings also support the notion that DNA methylation-driven genes are likely to be associated with clinical outcomes and can be utilized as potential biomarkers for predicting the prognosis of thyroid cancer.

## Conclusion

In conclusion, we screened DNA methylation-driven genes in thyroid cancer for the first time by using bioinformatics analysis from the TCGA database. A four-gene signature was constructed firstly by employing DNA methylation-driven genes, which served as an independent prognostic indicator for thyroid cancer. The results of our study may provide new method for the identification of thyroid cancer patients with clinical high-risk, and may open the way for the possible clinical application of methylation-driven genes.

## Data Availability Statement

All RNA-seq transcriptome data, DNA methylation data, and clinicopathological information are available in the TCGA database.

## Ethics Statement

The studies involving human participants were reviewed and approved by The First Affiliated Hospital of Zhejiang University. The patients/participants provided their written informed consent to participate in this study.

## Author Contributions

LL, LC, GH, QS, and JW conceived and designed the present study. LL, LC, and GH analyzed the data. LL and LC interpreted the data and wrote the manuscript. All authors read and approved the final manuscript.

## Conflict of Interest

The authors declare that the research was conducted in the absence of any commercial or financial relationships that could be construed as a potential conflict of interest.

## Footnotes

1 https://portal.gdc.cancer.gov/

2 https://david.ncifcrf.gov/

3 http://kobas.cbi.pku.edu.cn/

4 www.gsea-msigdb.org

## Supplementary Material

FIGURE S1 The partial enriched GO terms **(A–D)** in GSEA analysis.

FIGURE S2 Validation of DNA methylation-driven genes in thyroid cancer cells. **(A)** Relative expression of RDH5 in cells treated with 5-aza-2-deoxycytidine (5-aza); **(B)** Relative expression of TREM1 in cells treated with 5-aza; **(C)** Relative expression of BIRC7 in cells treated with 5-aza; **(D)** Relative expression of SLC26A7 in cells treated with 5-aza. ^∗^*p* < 0.05, ^∗∗^*p* < 0.01 and ^∗∗∗^*p* < 0.001.

TABLE S1 The results of univariate Cox analysis. HR, hazard ratio.

TABLE S2 The clinical information of the validated thyroid cancer cohort.
