# Supplementary figures and images for: Methylation-Driven Genes Identified as Novel Prognostic Indicators for Thyroid Carcinoma
Source: Front Genet. 2020 Mar 31;11:294. doi: 10.3389/fgene.2020.00294 (PMC7136565; doi:10.3389/fgene.2020.00294)

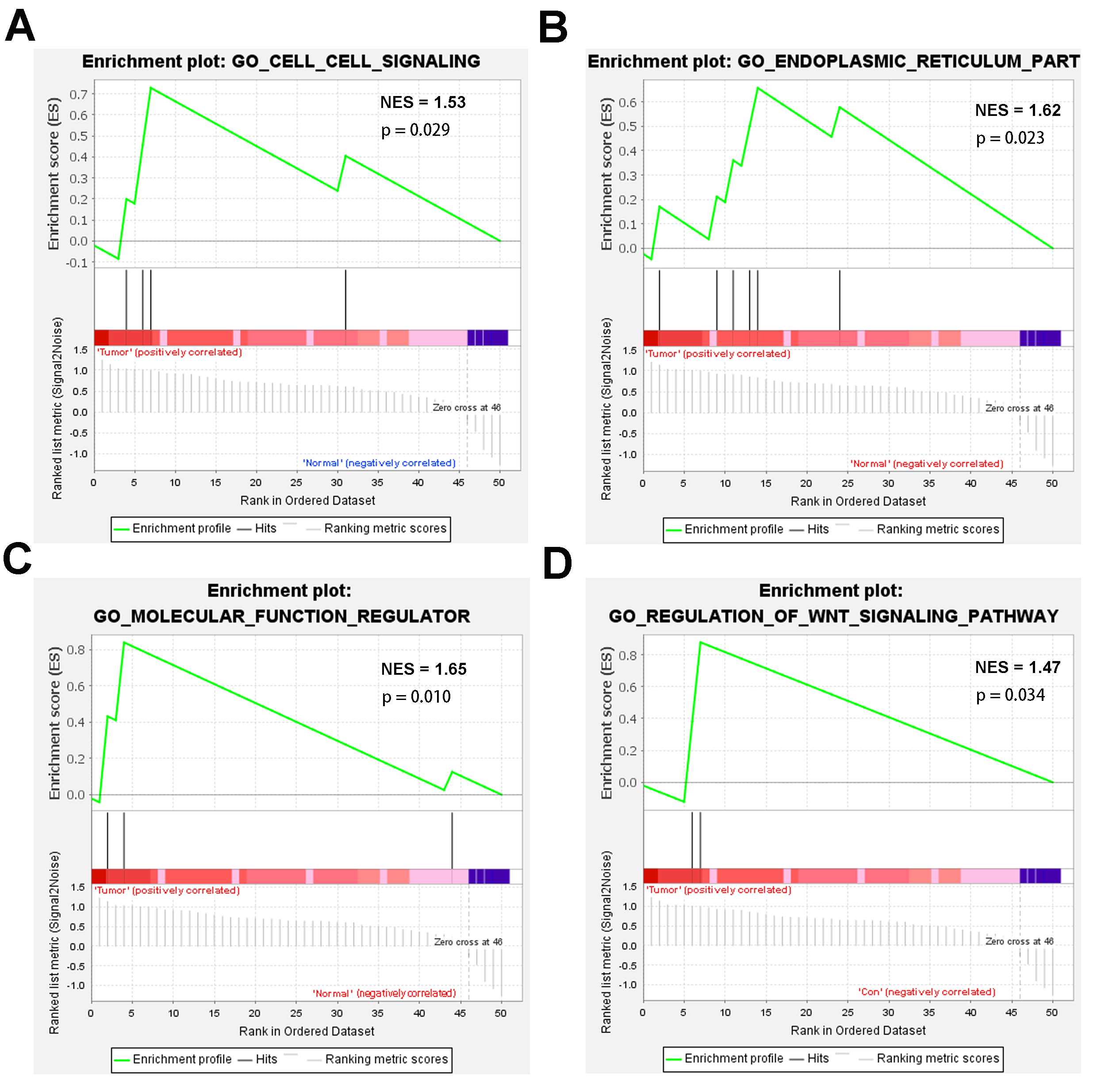

Supplement: FIGURE S1 — The partial enriched GO terms (A–D) in GSEA analysis. [file Image_1.TIF]

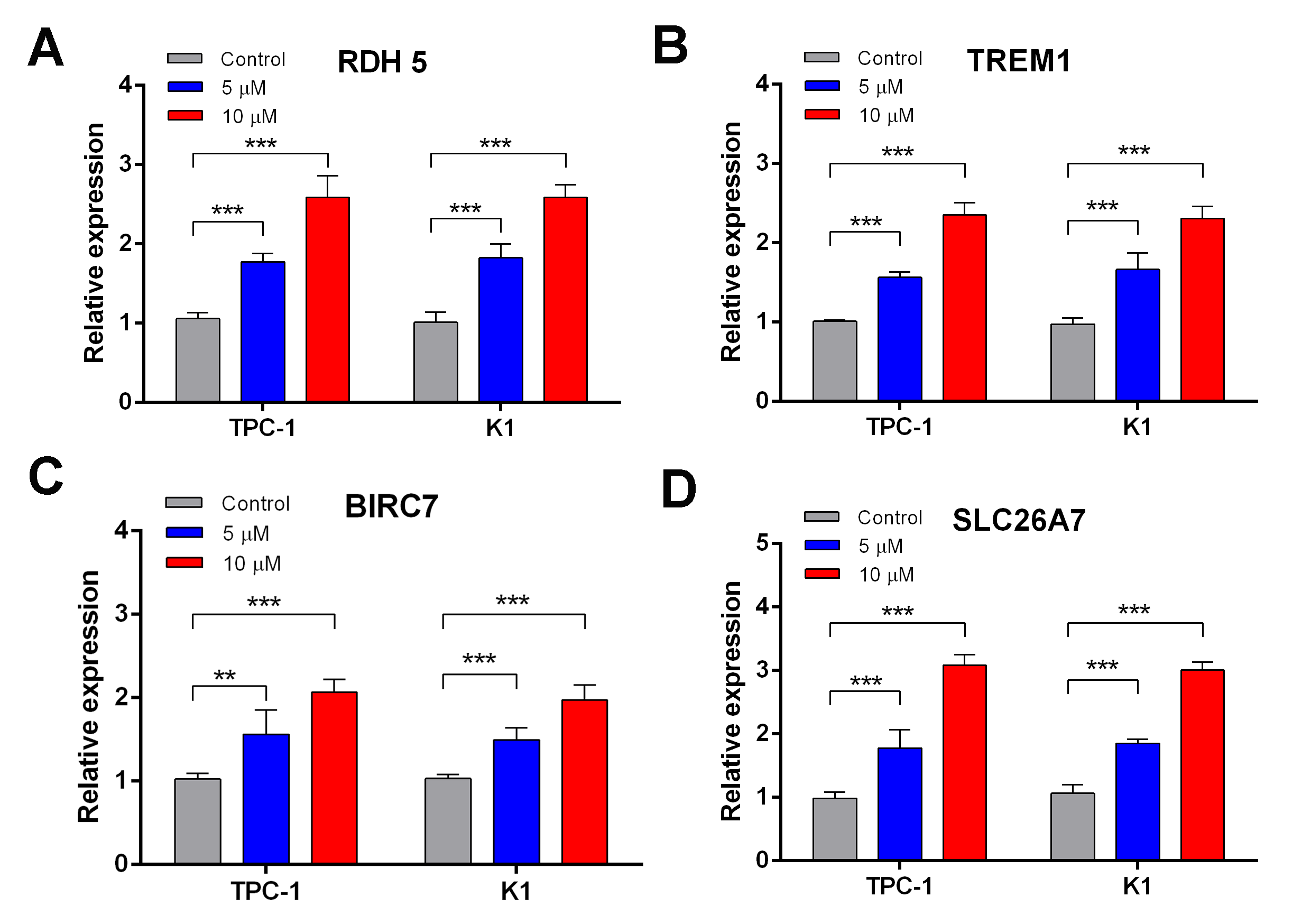

Supplement: FIGURE S2 — Validation of DNA methylation-driven genes in thyroid cancer cells. (A) Relative expression of RDH5 in cells treated with 5-aza-2-deoxycytidine (5-aza); (B) Relative expression of TREM1 in cells treated with 5-aza; (C) Relative expression of BIRC7 in cells treated with 5-aza; (D) Relative expression of SLC26A7 in cells treated with 5-aza. ∗p < 0.05, ∗∗p < 0.01 and ∗∗∗p < 0.001. [file Image_2.TIF]
