# Supplementary material for: Methylation-Driven Genes Identified as Novel Prognostic Indicators for Thyroid Carcinoma
Source: Front Genet. 2020 Mar 31;11:294. doi: 10.3389/fgene.2020.00294 (PMC7136565; doi:10.3389/fgene.2020.00294)
Supplement: TABLE S1 — The results of univariate Cox analysis. HR, hazard ratio. [file Table_1.docx]

**Supplementary Table 1** The results of univariate Cox analysis.

| genes | HR | 95% CI of HR | p value |
| --- | --- | --- | --- |
| LPAR5 | 0.927 | 0.883-0.974 | 0.002 |
| RDH5 | 0.573 | 0.364-0.901 | 0.016 |
| TREM1 | 1.102 | 1.010-1.203 | 0.030 |
| LIPH | 0.943 | 0.895-0.994 | 0.028 |
| CDH16 | 1.014 | 1.001-1.028 | 0.030 |
| BIRC7 | 1.012 | 1.002-1.022 | 0.018 |
| SLC26A7 | 1.013 | 1.005-1.021 | 0.002 |

Abbreviation: HR, hazard ratio.
