# Supplementary material for: Methylation-Driven Genes Identified as Novel Prognostic Indicators for Thyroid Carcinoma
Source: Front Genet. 2020 Mar 31;11:294. doi: 10.3389/fgene.2020.00294 (PMC7136565; doi:10.3389/fgene.2020.00294)
Supplement: TABLE S2 — The clinical information of the validated thyroid cancer cohort. [file Table_2.docx]

Supplementary Table 2. The clinical information of the validated thyroid cancer cohort

| Clinicopathological features | Number |
| --- | --- |
| Age |  |
| Mean (SD) | 40.72 (12.30) |
| Gender, n (%) |  |
| Male | 52 (26%) |
| Female | 148 (74%) |
| TNM stage |  |
| Stage I | 131 (65.5%) |
| Stage II | 17 (8.5%) |
| Stage III | 41 (20.5%) |
| Stage IV | 11 (5.5%) |
| T stage |  |
| T1 | 54 (27%) |
| T2 | 73 (36.5%) |
| T3 | 68 (34%) |
| T4 | 5 (2.5%) |
| N stage |  |
| N0 | 90 (45%) |
| N1 | 92 (46%) |
| N2 | 18 (9%) |
| M stage |  |
| M0 | 116 (58%) |
| M1 | 4 (2%) |
| MX | 80 (40%) |
|  |  |
